# Neuroprotective Mechanisms of *Red Algae*-Derived Bioactive Compounds in Alzheimer’s Disease: An Overview of Novel Insights

**DOI:** 10.3390/md23070274

**Published:** 2025-06-30

**Authors:** Tianzi Wang, Wenling Shi, Zijun Mao, Wei Xie, Guoqing Wan

**Affiliations:** 1 School of Chemistry and Chemical Engineering, Shanghai University of Engineering Science, Shanghai 201620, China; wtz5254011302@163.com; 2 School of Pharmacy, Shanghai University of Medicine and Health Sciences, Shanghai 201318, China; 18402109972@163.com (W.S.); mmcsjua@163.com (Z.M.)

**Keywords:** *Red algae*, Alzheimer’s disease (AD), neuroprotection, extraction methods

## Abstract

Alzheimer’s disease (AD) is characterized by β-amyloid plaques, neurofibrillary tangles, neuroinflammation, and oxidative stress—pathological features that pose significant challenges for the development of therapeutic interventions. Given these challenges, this review comprehensively evaluates the neuroprotective mechanisms of bioactive compounds derived from *red algae*, including polysaccharides and phycobiliproteins, which are considered a promising source of natural therapeutics for AD. *Red algal* constituents exhibit neuroprotective activities through multiple mechanisms. Sulfated polysaccharides (e.g., carrageenan, porphyran) suppress NF-κB-mediated neuroinflammation, modulate mitochondrial function, and enhance brain-derived neurotrophic factor (BDNF) expression. Phycobiliproteins (phycoerythrin, phycocyanin) and peptides derived from their degradation scavenge reactive oxygen species (ROS) and activate antioxidant pathways (e.g., Nrf2/HO-1), thus mitigating oxidative damage. Carotenoids (lutein, zeaxanthin) improve cognitive function through the inhibition of acetylcholinesterase and pro-inflammatory cytokines (TNF-α, IL-1β), while phenolic compounds (bromophenols, diphlorethol) provide protection by targeting multiple pathways involved in dopaminergic system modulation and Nrf2 pathway activation. Emerging extraction technologies—including microwave- and enzyme-assisted methods—have been shown to optimize the yield and maintain the bioactivity of these compounds. However, the precise identification of molecular targets and the standardization of extraction techniques remain critical research priorities. Overall, *red algae*-derived compounds hold significant potential for multi-mechanism AD interventions, providing novel insights for the development of therapeutic strategies with low toxicity.

## 1. Introduction

Alzheimer’s disease is a typical neurodegenerative disease that involves the slow erosion of cognitive and memory functions in patients. It is characterized by a progressive loss of neuronal structure and function, especially neuronal degeneration. Certain neuroprotective treatments have been associated with the amelioration of metabolic disturbances in the hippocampus and cerebral cortex [1]. Amyloid-beta (Aβ) accumulation and tau protein hyperphosphorylation are typical pathological features of AD, leading to the formation of senile plaques and neurofibrillary tangles; these are accompanied by certain secondary pathological changes such as neuroinflammation, synaptic dysfunction, and oxidative stress. Due to increasing socialization, population densification, and aging, the prevalence of AD and associated diseases is increasing: it is expected that, by the middle of the 21st century, the number of AD patients may double compared to current figures, resulting in exponential increases in the costs of healthcare, nursing, and patient care systems, posing additional challenges due to various risk factors [2,3].

Therapeutic agents for the treatment of AD currently available on the market include acetylcholinesterase inhibitors, NMDA receptor antagonists, new monoclonal antibody drugs, or combinations of these drugs [4]. These treatments generally relieve the symptoms of AD, but cannot completely eradicate or cure this disease. Furthermore, the use of these drugs can lead to significant side effects, including gastrointestinal, cardiovascular, and other adverse reactions, resulting in poor tolerance by patients. Therefore, the search for new therapeutic drugs of high quality and with low side effects is essential [5]. This search has led to increasing interest in other directions, especially natural medicines. In this context, seaweeds have received significant attention due to their multiple medicinal values, rich nutritional content, and metabolites with health-improving properties.

In marine ecosystems, *red algae* are ancient eukaryotic organisms classified under the group of seaweeds according to their pigmentation, and are considered important primary producers. They are rich in bioactive components, and primary and secondary metabolites derived from these species—including sulphated polysaccharides, pigments, phenolic compounds, and so on—play important roles in the nervous system and immune system, as well as possessing anti-inflammatory activities. There are about 7000 known species of *red algae* in existence, which occupy an important position in the fields of nutrition and medicine [6]. Algae show different adaptive abilities in different geogenic environments, including changes in appearance (e.g., color and size) and intrinsic properties (e.g., bioactivity and biochemical composition). *Red algae* have been reported to show greater adaptability to extreme environments compared to the other two types of algae classified according to their color; namely, brown algae and green algae. They can survive in polar pH conditions and at relatively high temperatures [7], and may generate metabolites with enhanced bioactivity and produce high-quality polymers with biological activity. *Red algae* can be divided into several economically important taxa: Chondrus species, for instance, are supported by substantial in vivo and in vitro data proving their potential anti-inflammatory, antioxidant, and neuroprotective properties; they are also a major raw material for carrageenan production [8]. Porphyra is one of the most-cultured genera of *red algae*, members of which typically contain large amounts of proteins and carbohydrates; as such, they are not only potentially economically beneficial, but also a source of numerous bioactive compounds with useful properties [9]. Gelidium amansii and Gracilaria are the main raw materials used for agarose production [10], both of which exhibit multifaceted neurotrophic activities [11]. As a result, *red algae* have received increasing attention and have served as the subject of several neuroprotective studies on AD. Most red algal extracts possess neuroprotective effects due to their anti-inflammatory and antioxidant properties; for example, carotenoids and phenolic compounds can protect neuronal cells due to their ability to scavenge free radicals, thus improving cognitive function and thereby reducing the risk of neurodegenerative diseases. In addition, lectins and mycosporine-like amino acids (MAAs), which have been reported in recent studies on *red algae* extracts, have shown potential to support brain health and cognitive function, further enhancing their neuroprotective effects.

In recent years, the development and application of algae-derived bioactive compounds has become an important direction in AD neuroprotection research. In the early stage of research, the main focus is on the extraction and functional evaluation of active ingredients from macroalgae such as *red algae*, with the selection and optimization of extraction methods directly determining the yield and quality of the extracted compounds. Regarding the systematic comparison between traditional solvent extraction methods and modern extraction techniques such as enzyme-assisted extraction and supercritical CO_2_ fluid extraction (the latter of which can better retain the biological activity of the extract), this review provides a comprehensive overview of the utilized extraction methods, as well as comprehensively elucidating the mechanistic features deriving from the extracted biochemical structures, in terms of their neuroprotective effects exerted against AD [12,13].

## 2. The Primary Neuroprotective Compounds of Red Algae and Their Characteristics

**Figure 1:**
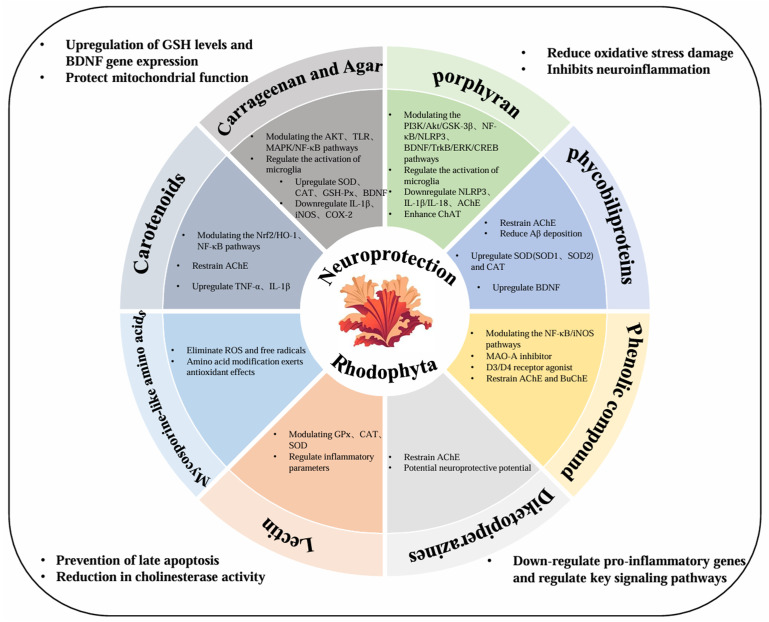
The main neuroprotective active components in *red algae* and their neuroprotective mechanisms.

As an important marine biological resource, *red algae* contain various systems of bioactive substance components, allowing them to produce abundant metabolites in extreme intertidal environments characterized by conditions such as high salt and strong UV [14]. Furthermore, their unique secondary metabolites constitute an all-encompassing system of neuroprotective active components [15]. These active components—including sulfated polysaccharides, algal bile proteins, phenolic compounds, and so on—exhibit systematic neuroprotective efficacy in multiple pathological aspects of Alzheimer’s disease, including oxidative damage, neuroinflammation, protein homeostatic imbalance, and synaptic dysfunction, thus providing a rich material foundation and theoretical basis for the development of novel marine-derived neuroprotective agents [16]. Figure 1 depicts the biological active substances present in *red algae* and their corresponding effects involved in neuroprotection against AD. 

### 2.1. Sulphated Polysaccharides (SPs)

Most of the polysaccharides in *red algae* exist in the cell wall, presenting an obvious hierarchical distribution. The sulfate-modified galactosaccharides carrageenan and agar are the carbohydrate constituents of *red algae* [17]; these are followed by heteropolysaccharides, with a more complex structure but relatively low content. In terms of neuroprotection, specific species are rich in porphyrin polysaccharides, typically based on specific structural features; as is generally the case regarding the diversity of bioactive compounds in *red algae* [18].

#### 2.1.1. Carrageenan and Agar

**Figure 2:**
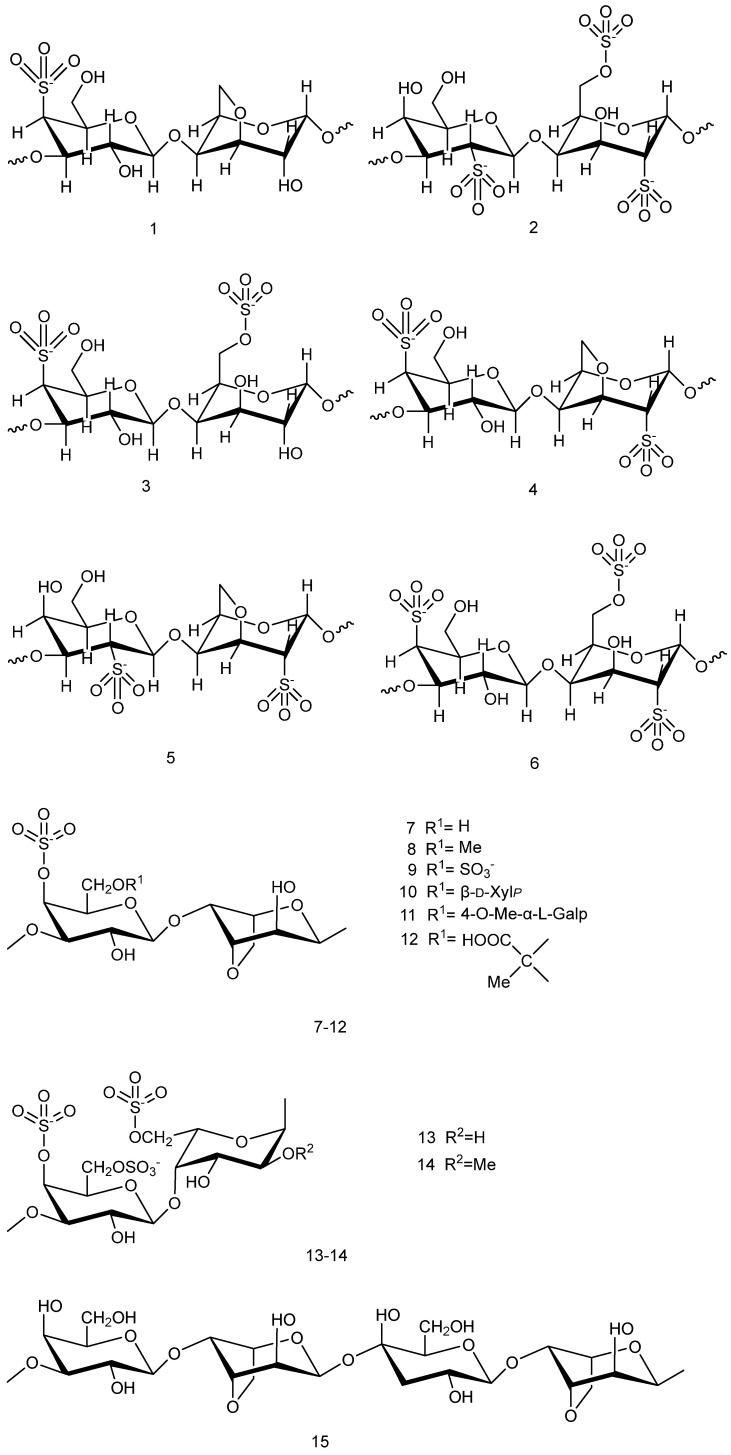
Chemical structures of different types of carrageenans and agars.

There are two main types of sulfated polysaccharides in red algae with neuroprotective effects; namely, carrageenan and agar, which are part of the compounds collectively referred to as phycobiliproteins. The backbone of the main chain of such polysaccharides mainly consists of D-galactose and 3,6-endo-l-galactose with alternating glycosidic bonds. They are characterized by highly complex structures, with structural diversity mainly reflected in the number and position of -OSO_3_ groups [19]. In general, the higher the content of sulfate groups and the smaller the relative molecular mass, the better the biological activity of the corresponding polysaccharide. Polysaccharides with small molecular weights also tend to possess better antioxidant activity [20,21]. Figure 2(**1**–**6**) show the different configurations of carrageenan extracted from *red algae* [22], while Figure 2(**7**–**15**) show the different structures of agar extracted from *red algae* [17,23]. The structural complexity of these compounds directly determines their unique biological activities and functional properties, leading to multifaceted neuroprotective effects.

**Figure 3:**
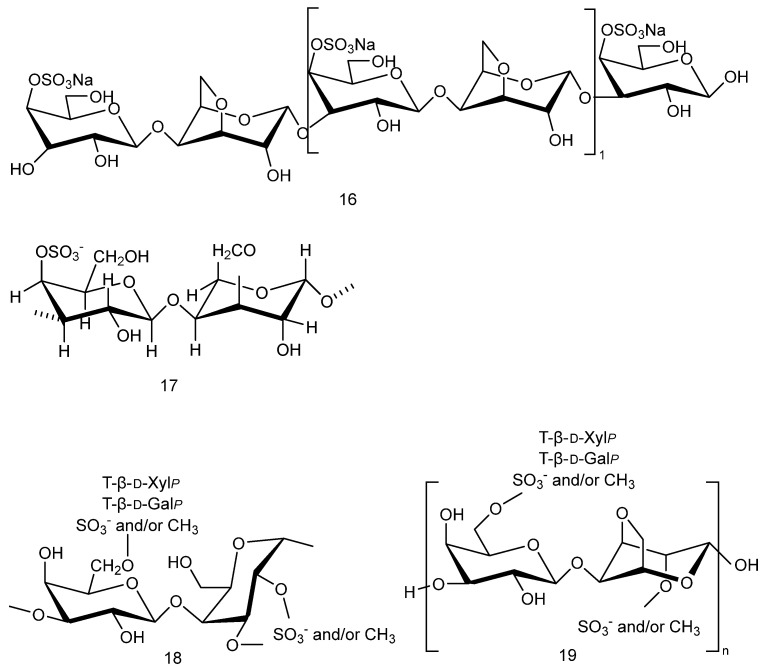
Structural formulas of sulfated polysaccharides with neuroprotective effects extracted from *red algae*.

Figure 3(**16**) shows the structural formula of a κ-carrageenan-derived pentasaccharide, which has been shown to attenuate Aβ25-35-induced neuroblastoma cytotoxicity and has potential therapeutic for AD [24]. Figure 3(**17**) shows an extracted κ- keratan gum enzymatic oligosaccharide KOS, which can protect nerves by inhibiting microglial hyperactivation [25]; in particular, microglia are significantly associated with AD [26]. Figure 3(**18**,**19**) show a sulfated xylogalactan (JASX) extracted from calcareous red seaweed, which has been shown to possess potent antioxidant properties [27]. Table 1 lists the different experimental models of sulfated polysaccharides derived from different sources of red algae.

The functional state of mitochondria can (directly or indirectly) affect synaptic and neuron-related properties, thus playing an important role in neuroprotection. As the core hub of oxidative stress regulation and apoptosis pathways in neuronal cells, mitochondrial dysfunction can lead to neuronal damage or even death; as such, maintaining mitochondrial health is one of the key strategies for neuroprotection [28,29]. Sulfated polysaccharides, mycosporine-like amino acids (MAAs), and chlorophyll derivatives extracted from Porphyra tenera have been shown to exert neuroprotective effects in multiple ways: first, through the regulation of mitochondrial function and the TLR/NF-κB pathway, they alleviated PM2.5-induced cognitive dysfunction; and second, through the inhibition of JNK phosphorylation to reduce the generation of Aβ and the hyperphosphorylation of tau proteins, directly demonstrating the significant antioxidant, anti-inflammatory, and neuroprotective efficacies of these active ingredients [30]. After the treatment of damaged cells using Porphyra yezoensis polysaccharides, cell viability increased, the amount of reactive oxygen species decreased, the mitochondrial membrane potential decreased, and intracellular Ca^2+^ release was inhibited [31]. Pelvetia siliquosa polysaccharides, through their antioxidant properties and up-regulation of the antioxidant enzymes SOD and CAT, were demonstrated to improve lysosome, cell morphology, and cytoskeleton integrity, thus inhibiting the decrease in mitochondrial membrane potential [32].

Brain-derived neurotrophic factor (BDNF) is one of the most important neurotrophic factors in the central nervous system, which can play neuroprotective and reparative roles; notably, neuroinflammation in AD can be inhibited and cognitive deficits can be counteracted through up-regulation of the BDNF/TrkB mechanistic pathway [33]. As a sulfated polysaccharide derived from red algal extract, carrageenan can regulate the expression of BDNF, and some studies have explored the effects of different molecular weight λ-carrageenans on Aβ-induced memory impairment in a rat model of Alzheimer’s disease. It was found that 1% low-MW λ-carrageenan could significantly enhance the level of BDNF, consequently improving memory functions; meanwhile, the study further confirmed that the inhibition of acetylcholinesterase was closely related to the development of AD, providing new insight into the neuroprotective mechanism of λ-carrageenan [34].

**Table 1:** The influences of carrageenan and agar on different experimental models, emphasizing their anti-inflammatory, antioxidant, and neuroprotective effects.

Activities	Source	Test Models/Cell Lines	Pathway/Mechanism	Effect on Test Models/Cell Lines	Author’s Conclusions	References
Antioxidant activity	*Gracilaria birdiae*	Mice induced by CCL4; 3T3-L1 cell	Glutathione and catalase levels	Increased antioxidant capacity and effects on the levels of glutathione reductase and catalase.	It has antioxidant activity and protective effect.	[35]
Neuroprotectant activity	*Gracilaria gracilis*	HT-22 cell line	Inhibition of apoptosis, oxidative damage, and acetylcholinesterase activity	Increased the contents of antioxidant enzymes and glutathione; the activity of acetylcholinesterase in cells decreased after zinc treatment.	These polysaccharides are good therapeutic agents for protecting neuronal cells from zinc-induced Alzheimer’s disease.	[36]
	κ-Carrageenan	APP/PS1 transgenic mice	Inhibition of the excessive activation of microglia, thereby demonstrating neuroprotective effects	Alleviated clinical symptoms in AD mice, decreased the expression of inflammatory factors and inflammation-related proteins in brain tissue.	KOS can be used as a therapeutic drug for neurodegenerative diseases.	[25]
	*Hypnea musciformis*	SH-SY5Y cells	Antioxidant and neuroprotective activities	Neuroprotective effect on 6-hydroxydopamine-induced neurotoxicity in SH-SY5Y cells.	Hm-SP shows neuroprotective activities.	[37]
Anti-inflammatory activity	*Spyrida Species Red Seaweed*	Bovine serum albumin (BSA)	Significantly inhibited the denaturation of bovine serum albumin (BSA)	Increasing the concentration of the extract from 25 to 100 µg/mL led to an increase in the percentage of inhibited protein denaturation.	The result was statistically significantly different from that of aspirin. It can be used medicinally.	[38]
	*Gelidium pacificum Okamura*	In LPS-stimulated human monocytic (THP-1) cells	Significant reduction of NO production in LPS-treated cells	Suppressed the mRNA and protein expression of TLR-4, MyD88, and TRAF-6.	Reduced LPS-induced cell toxicity and presented an anti-inflammatory effect via the TLR4 signaling pathway.	[39]
	*Gelidium crinale*	Lipopolysaccharide (LPS)-induced oxidative stress	Lipopolysaccharide (LPS)-induced oxidative stress and inflammation in macrophages	GNP had fairly strong scavenging activities on ABTS, hydroxyl, and DPPH radicals and had Fe2+-chelating ability in a dose-dependent manner.	GNP may be a latent anti-inflammatory component in pharmaceutical and functional food industries.	[20]
	*Gracilaria caudate*	Male Swiss mice	Reductions in joint oedema, MPO activity cell influx, IL-1β, and NO levels	Improved neutrophil migration to inflamed tissue, inhibited hyper-nociception, oedema.	Treatment for arthritic inflammation.	[40]
	*Gracilaria lemaneiformis*	IEC-6 cells	Inhibition of LPS-induced NO, TNF-α, and IL-6 production in IEC-6 cells	S-PS fractions possess anti-inflammatory activity.	Modulates inflammation and auto-immune diseases.	[41]
	*Gelidium amansii*	Diets-induced obese (DIO) C57BL/6J mice	Increased levels of anti-inflammatory cytokine production and lipolysis protein	Achieved anti-inflammatory and lipolysis-promoting effects.	Improves health conditions related to inflammation.	[42]

Sulfated polysaccharides extracted from *red algae* have been shown to exert multi-mechanism anti-inflammatory effects, with their neuroprotective effects stemming from inflammatory responses (especially those relating to neuroinflammation), as well as the inhibition of LPS-induced expression of iNOS and COX-2 and the secretion of pro-inflammatory factors through regulation of the TLR4/NF-κB signaling pathway [43]. Through regulation of the NO pathway, peritoneal MPO activity and IL-1β levels can be reduced while elevating NO_3_/NO_2_ values, thereby inhibiting the inflammation mediated by neutrophil migration [44], showing the potential of certain compounds for development as novel anti-inflammatory therapeutic agents. κ-carrageenan oligosaccharides (KOS) effectively improved cognitive dysfunction in APP/PS1 transgenic AD model mice by inhibiting microglial overactivation, significantly reducing Aβ deposition and inflammatory factor levels in the brain. It was verified in vitro that this compound attenuates microglia-mediated neurotoxicity and that inhibition of microglia in the hippocampus can have an ameliorative effect on mood deficits in mice, thus exerting a neuroprotective mechanism [45]. From these experiments and findings, it can be concluded that KOS has the potential to modulate neuroinflammation, providing a basis for the development of novel therapeutic strategies for AD [25]. Regarding the neuroinflammatory mechanism of Carrageenophyte Kappaphycus malesianus, Nicole Jean-Yean Lai et al. explored the inhibitory effects of its different solvent extracts on LPS-induced neuroinflammation in BV2 microglia. From the results, it was found that its methanol extract effectively inhibited the AKT/NF-κB and ERK signaling pathways, as well as significantly reducing pro-inflammatory cytokine expression without cytotoxicity (cell survival remained good in the concentration range of 0.16–2.5 mg/mL), whereas the ethanol extract exhibited significant cytotoxicity at 5 mg/mL (survival decreased to 6.58%). These findings suggest that the *K. malesianus* methanolic extract exerts safe and effective anti-neuroinflammatory effects, mediated by the modulation of key inflammatory pathways [46].

Antioxidants exert neuroprotective effects through multiple synergistic mechanisms such as neutralizing reactive oxygen radicals, modulating key signaling pathways, and attenuating damage due to oxidative stress, forming a multi-level defense network. Sulfated polysaccharides and their degradation products derived from Grateloupia livida exhibited significant antioxidant activities. Firstly, the degradation conditions were optimized, yielding a microwave power of 250 W, temperature of 54 °C, H_2_O_2_ concentration of 23 mM, and time of 33 min. The degradation fraction (DGLP) obtained based on this optimal protocol showed a DPPH radical scavenging rate of 71.39 ± 0.17%, from which two sulfated polysaccharide fractions—DGLP-1 and DGLP-2—were further isolated and purified. The structural characterization and activity evaluation confirmed DGLP-2 to exhibit superior antioxidant properties—significantly stronger than those of DGLP-1—providing a scientific basis for the development of antioxidant components of sulfated polysaccharides derived from *red algae* [47]. This approach indirectly reflects the ability of natural antioxidants to neutralize endogenous free radicals in the nervous system, and is considered an important tool for the discovery of neuroprotective compounds. In a study of sulphated polysaccharides derived from Porphyra haitanensis and Porphyridium cruentu, the extracted sulphated polysaccharides showed excellent antioxidant activity in the 2,2-azino-bis-3-ethylbenzothiazoline-6-sulfonic acid (ABTS) radical assay, with high scavenging capacity of 53.16% at 2 mg/mL [48] and 2.23 ± 0.30 μmol TE g-1 DW, respectively [49]. Gracilaria lemaneiformis polysaccharides (GLPs)—which have been widely studied and reported, due to the geophysical qualities of the species—possess powerful antioxidant activity and properties that help to prevent oxidative stress-related diseases; for example, Wang et al. found that they decrease senescence-associated β-galactosidase activity and facilitate suppression of p21 and p53 gene expression [50]. Furthermore, He et al. systematically evaluated the antioxidant activities of different polysaccharides using a number of in vitro indices, such as their ABTS radical scavenging, hydroxyl radical scavenging, nitrite scavenging, and reducing abilities. The constitutive relationships between their antioxidant efficacy and chemical properties were analyzed in depth, which provided an important basis for further revealing the molecular mechanisms underlying the antioxidant effects of polysaccharides [51].

Sulfated polysaccharides can also potentially exert neuroprotective effects through synergistic anti-inflammatory and antioxidant activities. Gelidium crinale-derived sulfated polysaccharides (GNP) were shown to possess significant dual antioxidant and anti-inflammatory activities: experiments showed that GNP can effectively scavenge ABTS, hydroxyl, and DPPH free radicals and, at the same time, significantly inhibited LPS-induced inflammation by blocking the MAPK/NF-κB signaling pathway-related expression of iNOS and COX-2, as well as the production of pro-inflammatory factors [20]. Signaling cascades upstream and downstream of MAPK also play important roles in apoptosis [52]; in particular, normal apoptotic activity can be neuroprotective, whereas necrotic apoptosis exacerbates neuroinflammation and further damages neural structures [53]. In addition, the degradation products of sulfated polysaccharides also exert antioxidant effects through different mechanistic pathways. LMSG—the degradation product of sulfated polysaccharides from Gracilaria fisheri—significantly elevated the expression levels of the antioxidant genes CAT and SOD through activation of the Nrf-2/ARE signaling pathway, simultaneously enhancing the cellular activities of GSH, CAT, and SOD, thus demonstrating excellent free radical scavenging ability [54]. Gracilaria lemaneiformis polysaccharide (GLP) and its degradation products presented significantly enhanced total antioxidant capacity (T-AOC) and promoted the activities of key antioxidant enzymes (SOD, GSH-Px) [55]. Sulfated polysaccharides derived from Porphyra haitanensis effectively improved the oxidative stress status of aging mice when administered via intraperitoneal injection, significantly reducing the level of lipid peroxidation while, at the same time, enhancing the total antioxidant capacity and the activities of key antioxidant enzymes (SOD and GSH-Px), demonstrating significant anti-aging and antioxidant effects [56].

#### 2.1.2. Porphyran

Porphyritic polysaccharides—generally obtained from red seaweeds of the genus Porphyra—are a class of compounds with a conjugated macrocyclic structure composed of alternating 4-linked α-l-galactopyranose-6-sulfate (L6S) and 3-linked β-d-galactopyranose (G) residues [57]. As a treasure trove of marine bioactive molecules, *red algae* are endowed with natural porphyrin-like compounds that exert potential neuroprotective effects through unique pharmacological and biological functions.

**Figure 4:**
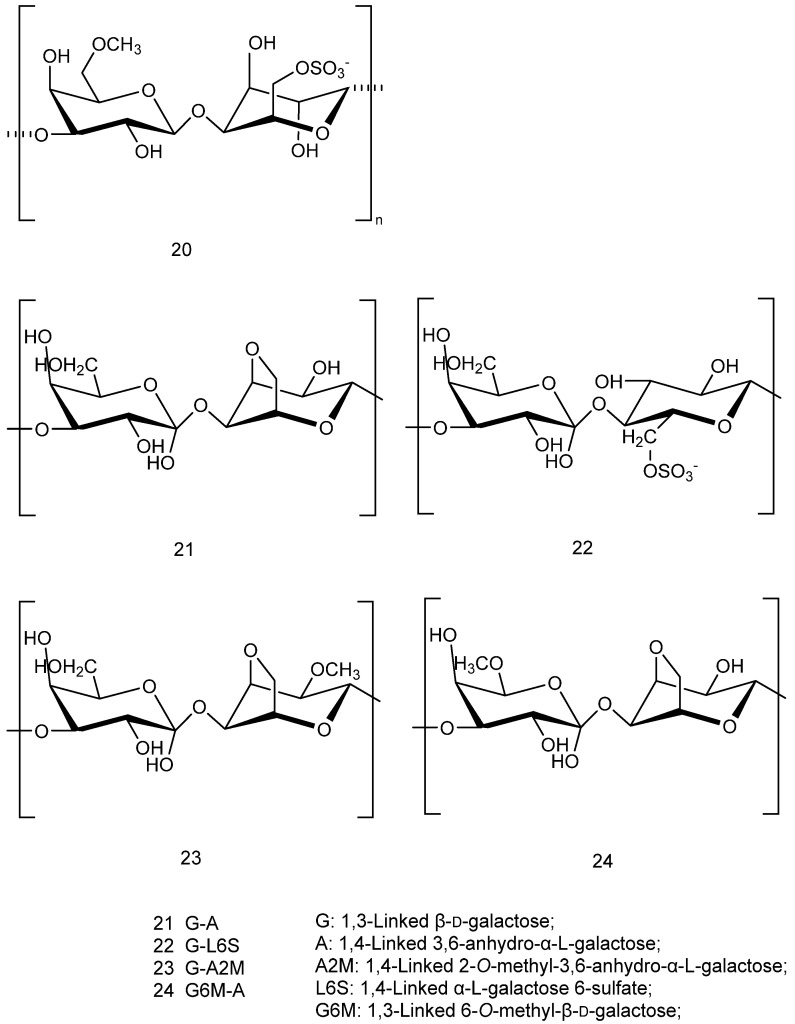
The chemical structures of porphyrins extracted from *red algae* [58,59].

Porphyran—a polysaccharide of red algal origin—has significant neuroprotective effects against cerebral ischemia/reperfusion (IR) injury. In vivo experiments have shown that a dose of 50 mg/kg of the drug improved motor function in gerbils and promoted neuronal survival in the hippocampal CAI region, with the main mechanism of regulation being inhibition of microglial activation and proliferation, as well as blocking the formation of the NLRP3 inflammasome pathway and downstream IL-1β/IL-18 release, thus providing targeted neuroinflammatory effects [58,59]. Detailed information on the methods, dosages, and results of various activity experiments involving porphyran and its derivatives is summarized in Table 2. Second, porphyran significantly improved cognitive dysfunction in Aβ1-40-induced AD model animals, where the mechanism of action was determined as bidirectional regulation of the cholinergic system: both enhancing choline acetyltransferase (ChAT) activity to promote acetylcholine synthesis, as well as inhibiting acetylcholinesterase (AChE) activity to reduce its degradation, thus effectively elevating the level of acetylcholine in the brain and providing a new potential intervention strategy for the treatment of AD [60]. Figure 4 illustrates the chemical structures of porphyrins extracted and isolated from *red algae*.

Oligo-porphyran (OP) also exhibited significant neuroprotective effects in a mouse model of MPTP-induced disease: first, OP maintained the expression levels of key proteins (dopamine transporter and tyrosine hydroxylase) in dopaminergic neurons; second, it activated the PI3K/Akt/GSK-3β signaling pathway, modulated the Bax/Bcl-2 ratio, and inhibited caspase-3 and PARP activation, thus effectively protecting dopaminergic neurons and ameliorating motor dysfunction, indicating that OP shows high potential as a neuroprotective agent [66]. In studies, degraded porphyran reduced pro-inflammatory cytokine release and inhibited hippocampal microglial overactivation by inhibiting the NF-κB/NLRP3 signaling pathway, while long-term porphyrin treatment activated the BDNF/TrkB/ERK/CREB signaling pathway in hippocampal regions in a mouse model of chronic unpredictable mild stress (CUMS). These findings suggest that degraded porphyrin exerts neuroprotective effects through dual anti-inflammatory and neurotrophic mechanisms [70].

### 2.2. Pigments

**Figure 5:**
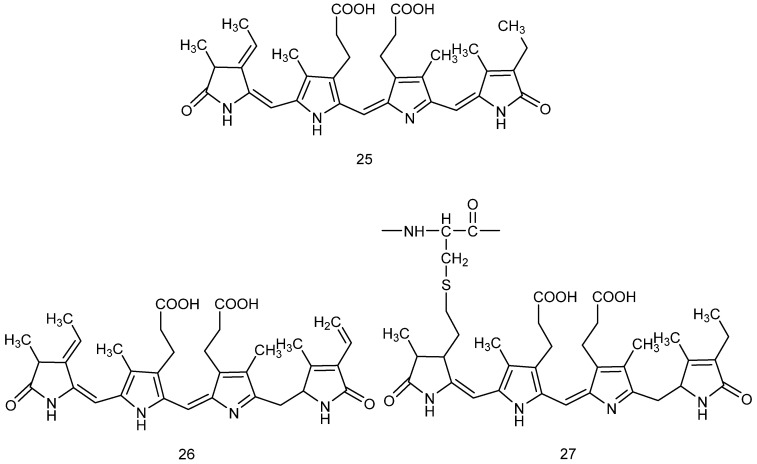
Chemical structures of phycobiliproteins.

The pigment composition in Rhodophyta species is key to their adaptation to underwater photosynthesis in marine ecosystems and is the main reason for their red to purplish coloration. Figure 5 depicts the chemical structures of various phycobilinproteins; in particular, Structure **25** represents the general structure of phycobilinproteins, while Structures **26** and **27** are the structures of phycoerythrin and phycocyanin, respectively. Red algal pigments (including phycobiliproteins, carotenoids, etc.) generally exert neuroprotective effects through antioxidant pathways. The pigment systems in *red algae* are significantly different from those in other algae, consisting of the major classes detailed below.

#### 2.2.1. Phycobiliproteins

Red algal phycobiliproteins (PBPs) are oligomers consisting of two subunits (α and β) which, generally in the form of a trimer (αβ)_3_ or a hexamer (αβ)_6_, are soluble in water (i.e., they are water-soluble pigmented proteins) [71]. Phycobiliproteins present significant absorption maxima at 498 nm, 565 nm, and 545 nm [72], which are part of red algal phycobilisomes as the core light-trapping pigments enabling photosynthesis in *red algae*. The three main classes include phycoerythrin (R-PE), phycocyanin (R-PC), and alloxyphycobiliprotein (APC). These proteins form a stable spatial structure through covalently bound chromophores and exhibit a rich range of colors, from light orange-red to deep blue-green in color. Forming an efficient light-trapping complex proteasome, the efficiency of light energy utilization is significantly enhanced through a special energy transfer mechanism, enabling *red algae* to adapt to diverse light environments in seawater of different depths [73]. Most strikingly, phycobiliproteins not only have excellent photophysical properties [74] but also show high potential in multiple biomedical fields, based on the characteristics detailed in Table 3.

Due to the special chemical structures and optical properties of phycobiliproteins, they have more comprehensive and specific roles in antioxidant systems and provide a basis for better treatment at the neuroprotective level. Reactive oxygen species (ROS) are associated with oxidative activity, and the antioxidant activities of PBPs derived from Pyropia yezoensis have been identified. The experiments were conducted on the basis of its structural features, leading to the extraction of 13 peptides (PBP1-13) derived from PBP. These phycobiliproteins (R-PE) effectively inhibited the generation of ROS in HepG2 cells, significantly reduced hydrogen peroxide-induced oxidative stress, and restored the expression level of superoxide dismutase (SOD), which confirmed their potential for the development of the red algal phycobiliprotein-derived peptides as novel antioxidants [75]. Protein dulse proteins extracted from *Palmaria* sp. have been shown to possess significant antioxidant activities, of which R-PE were the main active components. Functional analysis, conducted by preparing a recombinant PEβ subunit (rPEβ) and chromophore, revealed that the free radical scavenging activity of rPEβ was lower than that of the natural algal proteins; meanwhile, the chromophore demonstrated extremely strong radical scavenging ability in the ABTS assay. The cellular assay revealed that the chromophore could effectively protect SH-SY5Y cells against H_2_O_2_-induced damage. The results indicated that the antioxidant activities of red algal proteins mainly derive from their chromophore structure, providing a molecular basis for the development of novel neuroprotective agents [76].

In the course of neuroprotection research focused on phycobiliproteins, more in-depth excavation of the characteristics of phycoerythrins and phycocyanins from *red algae* based on its heat and acid resistance resulted in a better antioxidant effect through an improved research method [77]. R-Phycocyanin (R-PC) was isolated and purified from Porphyra haitanensis, and it was confirmed that both subunits had significant antioxidant activity: in vitro, it effectively scavenged free radicals and protected HUVEC cells from H_2_O_2_-induced apoptosis, with its mechanisms of action including up-regulation of SOD1, SOD2, and CAT expression; reduced ROS levels; and inhibition of lipid peroxidation, suggesting that R-PC exerts anti-aging and oxidative stress-protective effects in a multi-targeted manner [78]. Multi-targeted therapeutic activities are generally observed in herbal medicines or compounded tonics, as exemplified through the modulation of six core targets (MAPK8, CTNNB1, NFKB1, EGFR, BCL2, and NFE2L2) co-engaged by LJF in the treatment of AD [79].

With the deepening understanding of the molecular mechanisms underlying AD, researchers have found that selective inhibition of acetylcholinesterase (AChE) is a highly promising therapeutic strategy. Through effectively inhibiting AChE activity, the concentration of acetylcholine in the synaptic gap can be significantly increased, thereby improving the impaired cholinergic neurotransmission function. This mechanism not only directly targets the cholinergic system dysfunction that is characteristic of AD but, with further research, it has also been found that AChE inhibitors may exert neuroprotective effects through a variety of pathways, providing an important intervention target for AD treatment. Drugs developed based on this mechanism have become an important choice for the clinical treatment of AD, and the development of novel AChE inhibitors is an active field of research [80]. AChE inhibitors—which have effective inhibitory and neuroprotective effects against Aβ aggregation—inhibit AChE, reduce the phosphorylation of tau proteins at the S396 residue, and provide neuroprotective effects by rescuing abnormal neuronal morphology and improving cell viability [81].

**Figure 6:**
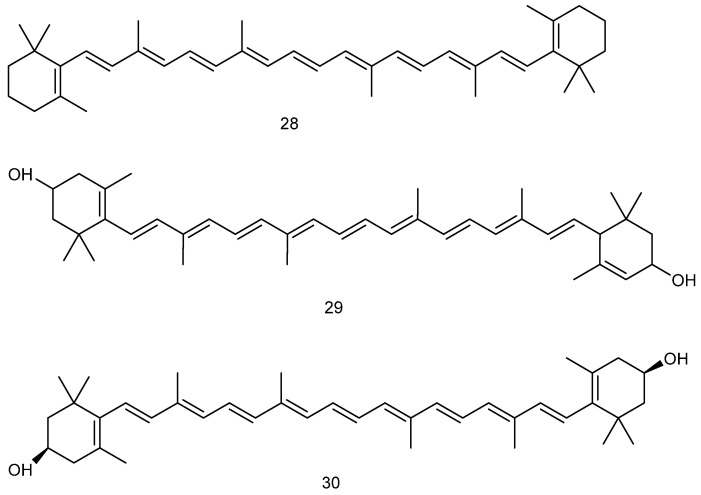
Chemical structures of carotenoids.

Studies on active peptides derived from *red algae* have revealed angiotensin-converting enzyme (ACE)- and dipeptidyl peptidase-IV (DPP-IV)-inhibitory activities and antioxidant properties. The enzymatic hydrolysis of Gracilariopsis chorda bile proteins by thermolysin led to the release of peptide fractions with multiple biological activities: three of the highly active peptide fractions showed significant ACE inhibition activity and a combined DPPH free radical scavenging ability. The neuroprotective mechanisms were as follows: ACE inhibition improves cerebral blood perfusion and may delay cognitive decline by blocking angiotensin II-mediated oxidative stress and neuroinflammation, decreasing β-amyloid deposition, and elevating BDNF expression. These findings provided motivation for the development of a dual neuroprotective–cardiovascular intervention strategy based on red algal peptides [82]. Windarto et al. used active peptides derived from *Acrochaetium* sp., which had been hydrolyzed using different protein hydrolases, as ACE inhibitors [83]. Water-soluble proteins (WSP) enriched in PE and PC were extracted from Grateloupia asiatica, then hydrolyzed by thermolysin to obtain hydrolysis products with angiotensin-converting enzyme (ACE)-inhibitory activity through a combination of computational analysis of the chloroplast genome and in vitro experiments. Finally, the compounds were evaluated by determining their IC50 value and ACE inhibitory activity in vitro [84]. Figure 6 depicts the chemical structures of carotenoids. Structure 28 represents the structure of β-carotene, while structures 29 and 30 correspond to lutein and zeaxanthin, respectively.

#### 2.2.2. Carotenoids

Carotenoids in *red algae* are composed of polyene chains with nine conjugated double bonds and terminal groups that constitute their basic structure, which are mainly divided into two categories: oxygen-free carotenoids (represented by α/β-carotene) and oxygen-containing derivatives (characterized by lutein and zeaxanthin). Carotenoids are characterized by a diversity of structural features and contain functional groups such as hydroxyl and carbonyl groups. These functional groups can form fatty acid esters, glycosides, and many other derivatives, and the differences in structure enrich their spectral properties and biological functions [85]. Through modification of the structure, stability, antioxidant activity, and related oxidation potentials can be improved through hydrogenation and ligand bonding [86]. The most studied carotenoids in *red algae* with demonstrated neuroprotective effects are lutein and zeaxanthin, which are isomers of each other. The specific properties of carotenoids extracted from *red algae* in terms of neuroprotection are as follows:

As typical carotenoids that exert neuroprotective effects, lutein and zeaxanthin extracted from *red algae*—which generally exert neuroprotective effects indirectly through anti-inflammatory or acetylcholinesterase inhibitory activities—provide a significant experimental basis for the prevention and treatment of AD; for example, regarding the modulation of AD-like pathologies by zeaxanthin and lutein in amyloid protein-β rat models. Compared with normal controls, AD model rats (AD-CON) exhibited significantly increased hippocampal Aβ deposition, impaired insulin signaling pathway (as evidenced by reduced Akt and GSK-3β phosphorylation levels), increased neuroinflammation, elevated acetylcholinesterase activity, and memory dysfunction. Meanwhile, zeaxanthin and lutein were effective in inhibiting acetylcholinesterase activity, decreasing lipid peroxidation levels, and decreasing the production of pro-inflammatory cytokines (e.g., TNF-α, IL-1β), which resulted in the amelioration of AD-related pathological features. These findings provide an experimental basis for the potential application of dietary carotenoids in the control of AD [87]. The anti-inflammatory effects associated with carotenoids have also been considered in the analysis of various inflammatory factors with respect to C-reactive protein; in particular, in a randomized controlled trial, carotenoid supplementation significantly reduced the levels of inflammatory markers. The overall analysis revealed strong effects on c-reactive protein (CRP) and IL-6, and the reduction of CRP by lutein/zeaxanthin was particularly significant (WMD: −0.30 mg/L, 95% CI: −0.45 to −0.15, *p* < 0.001), confirming the clear anti-inflammatory effects of these carotenoids [88]. In addition, β-carotene significantly reduced the levels of inflammatory mediators—including NO, PGE2, TNF-α, and IL-1β (*p* < 0.05)—and significantly reduced autophagy by up-regulating the autophagy marker LC3-II, while inhibiting the JAK2/STAT3, NF-κB, and JNK/p38 MAPK signaling pathways (*p* < 0.05) to exert anti-inflammatory effects [89].

The study of dual anti-inflammatory and antioxidant mechanisms could provide additional insights into neuroprotection in the context of AD. In a rigorously designed, double-blind, placebo-controlled clinical trial, daily supplementation with specific doses of lutein (10 mg), zeaxanthin (2 mg), and endocannabinoid zeaxanthin (10 mg) was effective in reducing key inflammatory markers (IL-1β, TNF-α) and oxidized low-density lipoprotein (OxLDL) levels [90]. In another study using a rat model of chronic excessive alcohol consumption (12 mL/kg-d for 12 weeks), rats in the alcohol model group showed a significant decrease in antioxidant capacity (decreased GSH-Px activity), increased oxidative damage (increased MDA levels), and enhanced inflammatory responses (IL-6, TNF-α levels), when compared to those supplemented with different doses of luteolin (12, 24, and 48 mg/kg-d). In particular, high-dose luteolin (48 mg/kg-d) enhanced antioxidant defense through activation of the Nrf2/HO-1 pathway, inhibited NF-κB-mediated inflammatory responses, and regulated apoptosis-related proteins (i.e., down-regulation of Bax, Cytc, and caspase-3, and up-regulation of Bcl-2). Up-regulation of the Nrf2/HO-1 signaling pathway can also inhibit ferroptosis in the hippocampus [91]. These findings confirm that luteolin can be considered useful, due to its dual antioxidant and anti-inflammatory properties [92].

### 2.3. Secondary Metabolites

Most of the compounds extracted from *red algae* need to be metabolized to improve their bioactivity. In terms of metabolites that may have better bioavailability in vivo, those derived from ASIV inhibited the expression of relevant proteins, down-regulated TNF-α, and inhibited the migration and proliferation of microglial cells, indicating therapeutic potential for neurological disorders [93]. Although carrageenan, agar, phycobiliproteins, and similar compounds are generally used as primary metabolites derived from *red algae* for neuroprotection, *red algae* also synthesize products based on their secondary metabolism, such as phenolic compounds, Mycosporine-like amino acids (MAAs), and terpenoids [22].

#### 2.3.1. Phenolic Compounds

**Figure 7:**
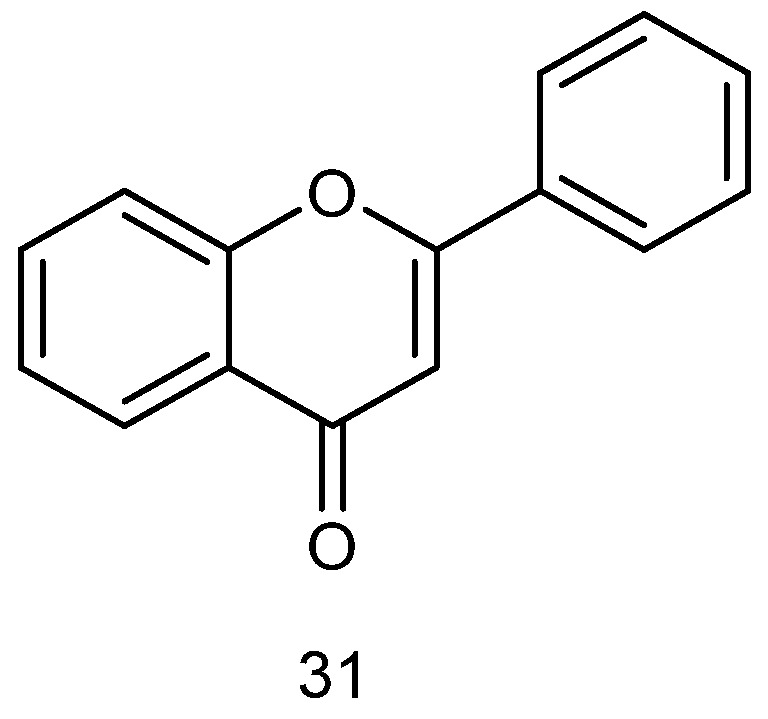
Chemical structure of phenolic compounds.

Phenolic compounds (Figure 7), due to the properties endowed by their functional groups, present significant scavenging ability in DPPH and ABTS free radical assays, indicating strong antioxidant capacity. Furthermore, they can inhibit the activity of AChE, thus possessing cytoprotective capacity [94]. The combined effect of these mechanisms indicate the great potential of red algal polyphenols in terms of neuroprotection.

Bromophenols from *red algae* exhibit unique neuroprotective potential. Bromophenols extracted from Symphyocladia latiuscula have been shown to act as monoamine oxidase A (MAO-A) inhibitors and dopamine D3/D4 receptor agonists, with intermediate selectivity for both, allowing for modulation of the dopaminergic system through a dual mechanism of action: MAO inhibition to reduce the degradation of dopamine and activation of D3/D4 receptors to enhance their signaling. In this way, they can be considered as novel marine drug candidate molecules for the treatment of AD with dual target characteristics, which thus may outperform traditional single-target drugs [95].

The mechanisms of cholinesterase inhibitors (ChEIs)—as standard therapeutic agents for AD and vascular dementia—are generally characterized on the basis of their structural properties [96,97]; for example, effectively blocking the degradation of synaptic gap ACh through selective inhibition of AChE and butyrylcholinesterase (BuChE) (inhibition constants Ki of 0.13–14.74 nM and 5.11–23.95 nM, respectively), thus improving cholinergic neurotransmission. Recent studies have revealed that synthetic natural bromophenols and their derivatives exhibit potent inhibitory effects on both key cholinesterases, with their excellent enzyme-inhibitory activities suggesting them as important lead compounds for the development of a new generation of multi-targeted neurotherapeutic drugs [98]. These unique bromophenol polyphenols possess not only significant acetylcholinesterase inhibitory activities (IC50 up to μg level), but can also effectively improve memory dysfunction [99].

**Figure 8:**
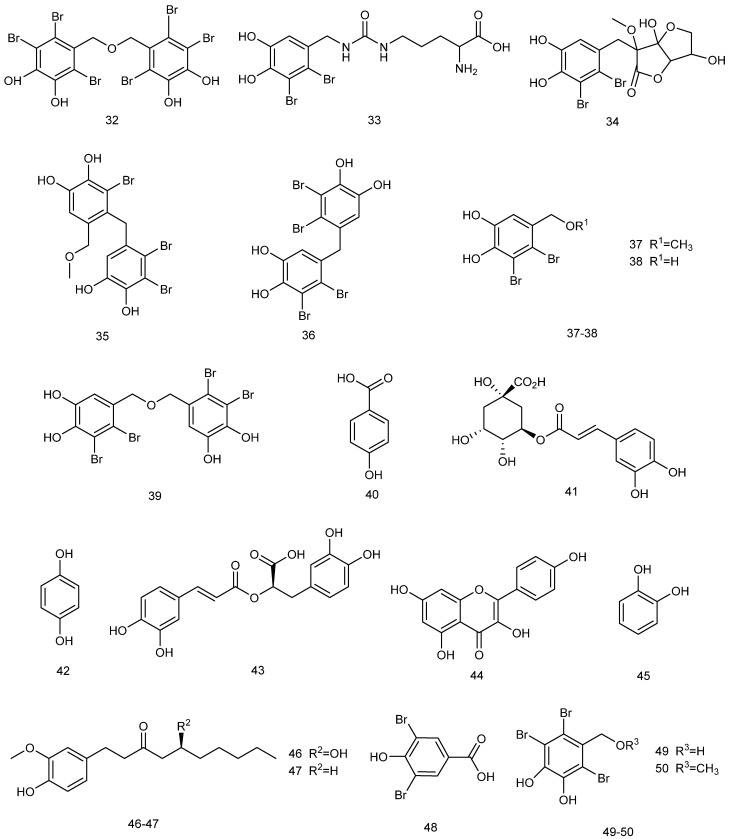
The structural formulas of phenolic compounds with neuroprotective effects extracted from *red algae*.

The natural bromophenol compound bis-diethyl ether (BTDE) extracted from marine *red algae* presents significant antioxidant activity. Experiments have shown that BTDE not only effectively scavenges ABTS free radicals, but also attenuates H_2_O_2_-induced ROS generation, reduces MDA levels, and improves redox status. Its mechanism of action is mainly mediated through selective activation of the AKT/Nrf_2_ pathway to up-regulate the expression of antioxidant enzymes (e.g., SOD), suggesting that BTDE—as a potential antioxidant functioning through the Nrf2-mediated pathway—can effectively protect HaCaT cells from oxidative damage [100]. Figure 8(**32**) shows the chemical structure of BTDE; (**33**–**39**) show those of *Bromophenolic* compounds isolated from *Vertebrata lanosa* [101]; (**40**–**45**) show those of *Bromophenolic* compounds extracted from *Galaxaura oblongata* [102]; (**46**–**48**) show those of phenolic secondary metabolites from the red alga Laurencia snackeyi [103]; and (**49**–**50**) show those of *bromophenolic* compounds derived from *Symphyocladia latiuscula* [95].

Phenolic compounds extracted from *red algae* generally exert potential neuroprotection from both inflammatory and antioxidant perspectives, and assays of free radical scavenging capacities (e.g., DPPH, ABTS, and FRAP reducing power) can be carried out to comprehensively determine the antioxidant activities of relevant extracts. The antioxidant activity of marine Gracilaria edulis extracts were systematically evaluated, and it was found that different solvent extracts presented different degrees of antioxidant properties in in vitro experiments: the ethyl acetate fraction exhibited prominent iron-reducing ability, iron ion chelation, and DPPH and ABTS radical scavenging activities; while the crude methanol extract showed excellent oxygen radical scavenging ability. These results reveal the value of Gracilaria edulis as a natural antioxidant source; in particular, its ethyl acetate fraction may contain a higher level of active antioxidant components [104]. Centroceras sp. exhibited the strongest antioxidant activity among the available test methods as it contained a significantly higher total phenolic content than other compared algal species, confirming the role of phenolic compounds as key antioxidant constituents, as well as indicating that phenolic content is significantly and positively correlated with antioxidant activity [105]. Evaluating the in vitro antioxidant activities of Gracilaria edulis and Hypnea valentiae polyphenol extracts, the total antioxidant activity of polyphenols amounted to 82.93 ± 0.48% in *G. edulis* and 78.12 ± 0.22% in *H. valentiae* as a result of DPPH, ABTS, hydroxyl, superoxide anion, and nitric oxide radical scavenging assays, as well as reducing power and hydrogen peroxide scavenging assays. Activity analysis revealed that these strong antioxidant properties mainly originated from the polyphenols in the extract—especially the flavonols and their glycoside derivatives identified therein—thus providing a key material basis for the development of *red algae* as sources of natural antioxidants [106]. In Jania rubens extract, total phenolic and flavonoid contents were determined as indicators of activity. The results revealed that the extract exhibited dose-dependent free radical scavenging activity (RSA percentage 10.3–50.2%) with an SC_50_ value of 3.87 mg/mL; it likewise exhibited significant reducing power in the FRAP assay (IC_50_ value of 46.84 ± 0.74 mg Trolox/g dry weight). These data confirm that J. rubens extract has definite antioxidant potential, and that its activity is closely related to the phenolic and flavonoid substances that it contains [107].

Phenolic substances extracted from *red algae* can also inhibit inflammatory responses and exert anti-inflammatory effects in neural pathways through modulation of the NF-κB/iNOS signaling pathway. The crude extract of the red alga Porphyra dentata was prepared via methanol extraction, and the main phenolic components—including catechol, rutin, and hesperidin—were identified using the HPLC-ESI-MS/UV coupling technique. In an LPS-induced RAW 264.7 macrophage inflammation model, the crude extract and its catechol and rutin components significantly inhibited NO production by blocking NF-κB enhancer activity and iNOS promoter activation. These results suggest that the anti-inflammatory effect of the *red alga P. dentata* can be mainly attributed to its polyphenols (e.g., catechol and rutin) [108].

#### 2.3.2. Mycosporine-like Amino Acids

**Figure 9:**
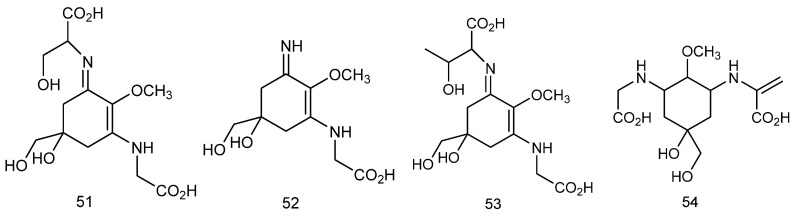
The chemical structures of MAAs extracted from *red algae*.

Mycosporine-like amino acids (MAAs) are a class of naturally occurring UV-absorbing compounds produced by *red algae* under stress conditions with unique dual bioactivities; namely, UV-absorbing secondary metabolites with antioxidant and photoprotective abilities [109]. Their characteristic structure contains a cycloheximide core skeleton modified by amino acid side-chains such as glutamine, glutamic acid, or threonine, which help them to exert better antioxidant and other bioactivities [110]. *Red algae* are considered to be the most productive source of MAAs. The structures of MAAs derived from red seaweeds are presented in Figure 9 [111]: (**51**–**54**) represent shinorine, palythine, porphyra-334, and palythenic acid, respectively.

Existing studies have not directly demonstrated the direct effects of MAAs extracted from *red algae* on neuroprotection in AD, but have demonstrated their potential therapeutic prospects based on neuroprotection starting from antioxidant defense mechanisms. The antioxidant activities of MAAs in different pH environments have been evaluated using ABTS and FRAP assay systems, and the experimental results showed that the antioxidant capacity of MAAs is pH-dependent, possessing the most excellent radical scavenging ability and the highest reducing power under alkaline conditions [112]. This may be related to the fact that deprotonation in alkaline environments enhances the electron delocalization ability of conjugated systems. Under conventional conditions, the ABTS radical scavenging rate and FRAP reducing power of MAAs in acidic environments were significantly lower than those under neutral and alkaline conditions; however, it was unexpectedly found that the antioxidant activity of heat-treated MAA crude extracts were enhanced in acidic environments [113].

### 2.4. Other Neuroprotective Compounds

#### 2.4.1. Lectin

Lectins are non-immunogenic sugar-binding proteins with highly diverse structures that are widely found in plants, animals, and microorganisms. In general, sugar chain structures at the cell surface can bind to lectins, which act to trigger a variety of biological activities [114]. Existing lectins targeting neuroprotection in AD provide various research directions and advances; for example, galactoglucan lectin-3 can modulate microglial activation to participate in the treatment of AD [115], while Canavalia ensiformis lectin (ConA) has been demonstrated to possess neuroprotective potential through the mediation of glutamatergic excitotoxicity [116]. Lectins extracted from *red algae* are generally oriented in the general direction of anti-inflammatory and antioxidant use for the time being and have considerable potential in this area. Red algal lectins are a group of biologically active proteins isolated from *red algae* that bind specifically to carbohydrates. Lectins isolated from the red alga Amansia multifida Lamouroux were found to modulate inflammatory parameters such as oxidative stress, pro-inflammatory cytokines, and leukocyte migration [117]. Their anti-inflammatory effects were assessed through the measurement of peroxidase activity, leukocyte and neutrophil migration, and quantification of cytokines in peritonitis models by Fontenelle et al. [118], and their antioxidant effects by assessing the enzymatic levels of glutathione peroxidase (GPx), catalase (CAT), and superoxide dismutase (SOD) [119].

#### 2.4.2. Diketopiperazines

**Figure 10:**
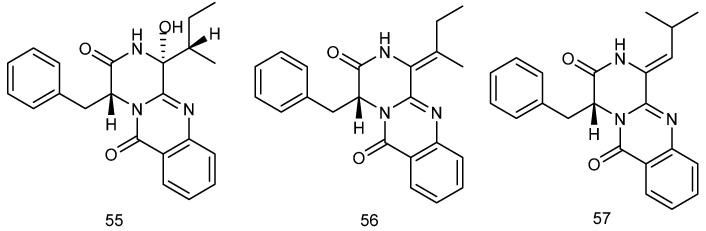
The chemical structures of diketopiperazines extracted from *red algae*.

Diketopiperazines (DKPs) are some of structurally simplest cyclic dipeptides, which have been widely extracted and isolated from *red algae* and are formed by the condensation of two amino acid molecules. DKPs have a unique rigid cyclic structure and diverse pharmacological activities, and have shown broad application prospects in the field of drug discovery and development. It is an important class of biologically active natural products [120], and *red algae* is one of the important sources of DKPs. Figure 10 shows the chemical structures of some diketopiperazines extracted from *red algae*, which have shown inhibitory activities against angiotensin-converting enzyme (ACE) [121]. Over the past decade, diketopiperazines have been shown to exert more and more neuroprotective effects through their own and amino acid modifications, leading to the investigation of potentially neuroprotective concentrations of histidine–proline DKP isomers as candidates for the treatment of AD [122]. Designing diketopiperazine derivative scaffolds with AChE, MMP2 inhibition, and strong blood–brain barrier penetration ability is expected to support AD treatment development [123]. Diketopiperazines derived from *red algae* have been shown to offer high therapeutic potential in AD and general neuroprotective studies.

#### 2.4.3. Homotaurine

Homotaurine (tramiprosate) is a naturally occurring sulfamic acid analog which is widely found in a variety of marine *red algae*. It has been shown that this substance tends to exert neuroprotective effects via multiple mechanisms of action: through specific inhibition of β-amyloid (Aβ) aggregation; activation of the γ-aminobutyric acid type a receptor (GABA_a receptor) [124]; and disrupting the interaction between PSD95 and nNOS, thereby inhibiting nNOS translocation [125]. These properties have been validated in several in vitro and in vivo experiments, confirming its significant neuroprotective potential.

It has also been shown that treatment with homotaurine significantly reduces reactive oxygen species (ROS) levels, enhances mesenchymal stem cell (MSC) viability, and maintains cellular homeostasis through the regulation of key stress-responsive proteins such as sestrin 1 and p21. In addition, homotaurine up-regulated β-catenin expression and further reduced ROS levels, suggesting that it alleviates oxidative stress and signaling pathway abnormalities associated with neurodegenerative diseases [126]. In preclinical studies, homotaurine has also shown potential therapeutic value in the context of amnestic mild cognitive impairment (aMCI), possibly exerting neuroprotective effects by protecting hippocampal structure and improving situational memory function [127].

**Figure 11:**
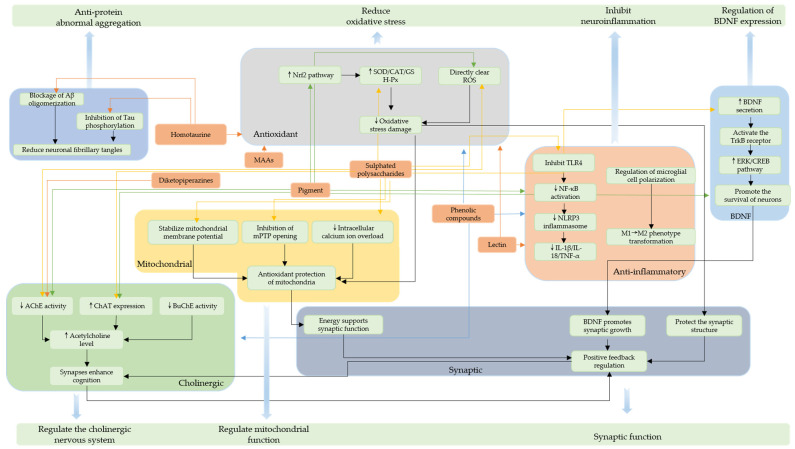
The main mechanism pathways of compounds derived from *red algae* in the context of neuroprotection.

Figure 11 shows the main mechanistic pathways of *red algae*-derived compounds at the neuroprotective level. These pathways are mainly characterized in terms of anti-inflammatory, antioxidant, mitochondrial, BDNF, synaptic, and cholinergic systems, based on which various neuroprotective effects are exerted, which may have significance in the context of AD. In particular, yellow lines represent the process pathways through which sulphated polysaccharides exert neuroprotection, green and blue represent the mechanistic processes of pigment and phenolic compounds, other classes of compounds are indicated in orange, and the linkages within or between modules are indicated in black.

**Figure 12:**
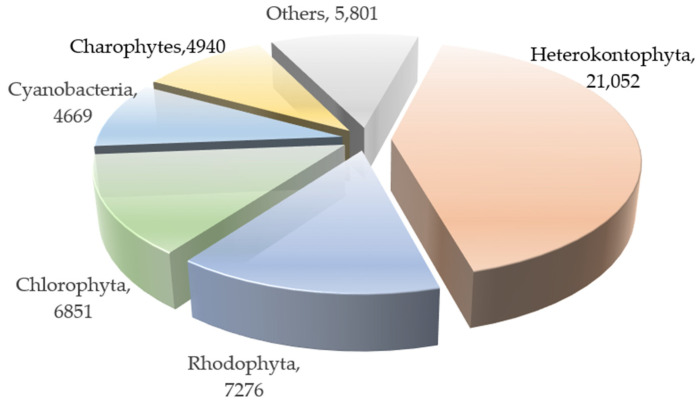
The proportion distribution of *red algae* and other major algae.

**Figure 13:**
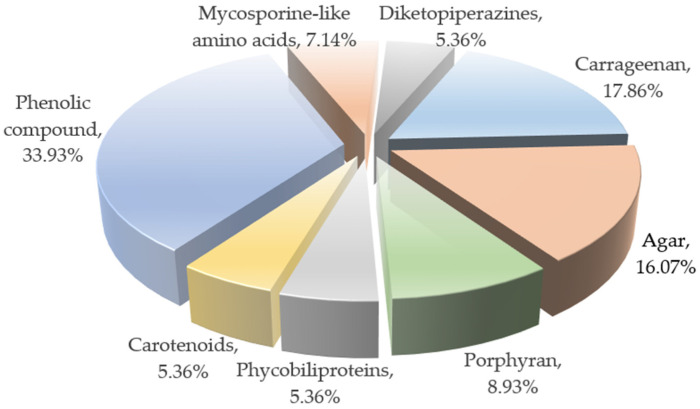
The proportions of structural categories known for possessing neuroprotective compounds isolated from *red algae* over the past decade.

This review summarizes the structures of compounds extracted and isolated from *red algae* presenting neuroprotective effects, as reported between 2015 and 2025, including sulfated polysaccharides, pigments, secondary metabolites, and other components, based on the AlgaeBase database. Figure 12 illustrates the distribution of the extant *red algae* and other major algae, of which the most abundant algae are Heterokontophyta (21,052 species), Rhodophyta (7276 species), and Chlorophyta (6851 species) [128]; these algae play an increasingly important role, as the second largest population group in nature. Figure 13 demonstrates the percentage of the isolated structures with the neuroprotective activity from *red algae*, comprising a total of 46 isolated compound structures. Of these, phenolic compounds have the highest percentage, with 17 isolated structures accounting for 36.96%, followed by carrageenan, porphyrin, and other sulfated polysaccharide compounds.

## 3. Extraction of Neuroprotective Compounds from Red Seaweed

In the process of studying the mechanistic properties associated with neuroprotective compounds in *red algae*, compounds usually have a low number of side-effects. Compounds derived from *red algae*, including polyphenols, phycobiliproteins, polysaccharides, etc., have attracted significant attention due to their antioxidant, anti-amyloidosis, anticholinesterase, and anti-inflammatory properties, and effective methods for their extraction, isolation, and purification can increase the efficiency and yield of these compounds; for example, through the optimization of the extraction process, the extraction rate of MAAs from *red algae* has been significantly improved [129]. Suitable extraction methods allow the biological activity of the compounds to be retained, thus enhancing their application value in the fields of medicine and food.

Emerging technologies provide significant advantages regarding the extraction of *red algae* compounds, when compared to traditional extraction methods. For example, microwave-assisted hydrothermal extraction (MAHE), as a green and efficient carrageenan extraction technique, allows for high-purity extraction without relying on chemical reagents while maintain its excellent rheological properties [130]. In addition, ultrasound can produce synergistic effects when combined with other conventional primary extraction methods. It has been shown that the combined maceration-ultrasound extraction method performed optimally among the multiple extraction methods, achieving 77% and 93% extraction of R-PE and R-PC, respectively. It can efficiently extract algal bile proteins from the rigid biomass of *red algae* [131]. These emerging methods not only improve the extraction efficiency and purity of the resulting product, but also reduce solvent usage, energy consumption, and environmental pollution. Due to their higher selectivity and cost-effectiveness, these methods provide better solutions for the industrialized extraction and application of red algal compounds.

Polysaccharides are macromolecules polymerized from monosaccharides, with structural diversity and functional specificity due to their composition of multiple chains. Their difference in solubility is relatively large, generally speaking, with neutral polysaccharides requiring hot water to dissolve, and acidic polysaccharides needing cold water to dissolve. Agar, when formed into a gel, will become highly viscous and gelatinous. The pH or temperature-sensitive environmental responsiveness of polysaccharides in the face of a variety of extraction methods and conditions necessitates the selection of an appropriate extraction method in order to improve the yield and production efficiency [132,133]. In addition, some auxiliary extraction optimization methods, such as the use of a response surface methodology to optimize the conditions for extracting polysaccharides (e.g., in terms of extraction time, temperature, and mass-to-water ratio) not only can improve the yield of polysaccharides, but also helps to optimize the experimental process [134]. Table 4 summarizes the various extraction methods for polysaccharides in *red algae* which have demonstrated neuroprotective effects, in terms of the source, the type of polysaccharides extracted, the method of extraction, the yield, and the composition of monosaccharides.

Due to the neurotoxicity and genotoxicity of some artificial colors, there is a need to find harmless alternatives to natural colors that can scavenge hydroxyl ions and avoid lipid peroxidation reactions. Natural pigments are renewable and sustainable biological resources with low environmental impact, which can also be used in other fields such as the cosmetics and pharmaceutical industries. Studies have shown that natural pigments in marine algae are suitable alternatives to artificial colors [135]. The pigments in *red algae* mainly include phycobilins, chlorophyll a, chlorophyll d, lutein, and zeaxanthin. These pigments give *red algae* their distinctive red to purplish color. Phycoerythrin and phycocyanin are light-trapping pigment proteins which are unique to *red algae*, possessing unique optical and biological activities, including the ability to play anti-inflammatory and antioxidant neuroprotective roles through a variety of mechanistic pathways. There are various extraction methods for the pigments in *red algae*, each with its own advantages and disadvantages. These include traditional solvent extraction methods, which are simple but less efficient; ultrasound-assisted extraction methods, which have improved efficiency but require more complex equipment; and enzymatic extraction methods, which are mild but more costly. The selection of a suitable extraction method should be based on the species of *red algae*, the target pigments, and the purpose of extraction [13].

The efficient extraction of polyphenols from *red algae* poses a number of key challenges: first, their active components (such as phenolic acids, flavonoids and bromophenols) are not only structurally complex and diverse, but also exist in the algal body in the form of a bound state, forming stable complexes with cell wall polysaccharides and proteins; second, these compounds are extremely sensitive to light, heat, pH, and oxygen, and are highly susceptible to oxidative degradation during extraction, which may result in a significant reduction of their bioactivity. In addition, the large number of sulfated polysaccharides, phycoerythrins, and other interfering components contained in the red algal matrix may not only impede the penetration efficiency of the solvent, but can also cause serious co-extraction problems in the subsequent purification steps. To further complicate matters, significant synergistic or antagonistic effects among the components in red algal extracts lead to a lack of direct correlation between total phenolic content and antioxidant activity, which poses great difficulties in the accurate screening and standardized preparation of active ingredients. Table 5 lists the statistics relating to traditional and emerging extraction methods for red algal pigments and polyphenolic compounds [105,136].

- Water extraction;
- Acidic and alkaline extraction;
- Soaking-stirring method;
- Innovative techniques;
- Ultrasound-assisted extraction (UAE);
- Microwave-assisted extraction (MAE);
- Ultrasound-microwave assisted extraction (UMAE);
- Enzyme-assisted extraction (EAE).

**Table 4:** The extraction methods used for red macroalgae polysaccharides.

Source	Polysaccharide Type	Extraction Method	Yield	Monosaccharides Composition	Time (min)	Temperature (°C)	Water/Material Ratio (mL/g)	References
*Eucheuma gelatinae*	β-carrageenan	Maceration-stirred	87.56 ± 5.61(%)	Rhamnose, mannose, glucose, fucose, and xylose	115.35 min	82.35 °C	36.42 (*v*/*w*)	[137]
*Porphyra haitanensis*	Porphyra haitanensis polysaccharides (PHPs)	PHP, UHP-PHP, US-PHP, and M-PHP	71.33%	Galactose, mannose, glucose and xylose	120.00 min	90 °C	1:40 (*v*/*w*)	[138]
*Porphyridium purpureum*	Porphyridium purpureum polysaccharides	A novel three-step extraction strategy	75.20%	Galactose (39.58%), xylose (38.83%), and glucose (21.59%)	120.00 min	80 °C	1:50 (*v*/*v*)	[139]
*Porphyridium purpureum*	Porphyridium purpureum polysaccharides	Response surface methodology, microwave-assisted extraction	25.48%	Glucuronic acid (150 °C), fucose (90 °C)	45 min	87 °C	1:63 (g/mL)	[140]
*Porphyra haitanensis*	Porphyra haitanensis polysaccharides (PHPs)	Water extraction and alcohol precipitation methods, single-factor and Box-Behnken response surface methodologies	20.48%	Galactose, glucose, and fucose	180 min	80 °C	0.04	[141]
*Rhodymenia intricata*	Rhodymenia intricata polysaccharides	Ultrasound-assisted water extraction method	37.78 ± 0.15%	α-pyranose	95 min	80 °C	1:84 (g/mL)	[142]
*Gracilaria chouae*	Gracilaria chouae sulfated polysaccharides	Citric acid extraction and water extraction	22.85 ± 0.80% (CGCP), 27.4 ± 0.12% (WGCP)	Galactose, glucose, and xylose	120 min	100 °C	1:25 (*w*/*v*)	[143]
*Sarcopeltis skottsbergii*	Carrageenan	Microwave-assisted hydrothermal treatment	63–64%	Galactose (54.00 ± 0.50%), glucose (3.92 ± 0.41%)	5-7 min	110 °C, 160 °C	1:30 (*w*/*w*)	[130]
*Mastocarpus stellatus*	Hybrid carrageenans	Microwave hydrodiffusion and gravity (MHG)	37%	Galactose + xylose + mannose yielded close to 25%, followed by glucose 3.5%	120 min	90 °C	-	[144]
*Gracilaria lemaneiformis*	Agar	Traditional alkaline extraction	4%	Galactose, anhydro-L-galactose	180 min	90 °C	1:20 (*v*/*w*)	[145]
*Gracilaria lemaneiformis*	Agar	Enzyme-assisted extraction technology	24.70%	Galactose, anhydro-L-galactose	60 min	50 °C	1:20 (*v*/*w*)	[145]
*Gracilaria lemaneiformis*	Agar	Enzymatic extraction	3.40%	Galactose, anhydro-L-galactose	180 min	50 °C	1:20 (*v*/*w*)	[145]
*G. sesquipedale*	Agar	Subcritical water extraction, moderate electric fields, and a combination of both methods	-	Galactose, anhydro-L-galactose	95 °C for 180 min, 110 °C for 60 min, 130 °C for 9 min, and 140 °C for 1 s		1:30 (*w*/*v*)	[146]
*G. vermiculophylla*	Agar	Subcritical water extraction, moderate electric fields and a combination of both methods	-	Galactose, anhydro-L-galactose	85 °C for 120 min, 110 °C for 15 min, 120 °C for 5 min, and 125 °C for 1 min.		1:30 (*w*/*v*)	[146]
*Porphyra haitanensis*	Porphyra haitanensis polysaccharides (PHP)	Ultrasonic/microwave-assisted extraction (UMAE), response surface methodology to optimize	20.98%	Galactopyranose	29.64 min	79.94 °C	1:41.79 g/mL	[147]

**Table 5:** The extraction methods used for red macroalgae pigments and phenolic compounds.

Source	Type	Extraction Method	Total Content of Pigments (mg/100 g)	Yield	Solvent	Time (min)	Temperature (°C)	References
*Gelidium sesquipedale*	Phycobiliproteins	Traditional serial extraction (5 h)	147.3 ± 3.2	100%	Ethanol, water, and aqueous ethanol solutions with concentrations of 50% and 70%	300 min	RT or 40 °C	[148]
*Gelidium sesquipedale*	Phycobiliproteins	Ultrasound-assisted extraction (UAE) 10 min	54.7 ± 1.6	37%	-	10 min	RT or 40 °C	[148]
*Gelidium sesquipedale*	Phycobiliproteins	Ultrasound-assisted extraction (UAE) 15 min	54.1 ± 2.1	37%	-	15 min	RT or 40 °C	[148]
*Gelidium sesquipedale*	Phycobiliproteins	UAE15min + maceration (Mac) 45 min	72.4 ± 0.5	49%	-	60 min	RT or 40 °C	[148]
*Gelidium sesquipedale*	Phycobiliproteins	UAE15 min + maceration (Mac) 60 min	77.9 ± 1.6	53%	-	75 min	RT or 40 °C	[148]
*Grateloupia turuturu*	R-phycoerythrin (R-PE)	Ultrasound-assisted enzymatic hydrolysis (UAEH)	4.28 ± 0.09 mg/g dry weight (dw) at 180 min	-	-	180 min	20 °C	[149]
*Porphyridium purpureum*	Phycoerythrin	Deep eutectic solvent (DES)-based ultrasound-assisted extraction (UAE)	-	-	DES or water	10 min	25 ± 1 °C	[150]
*Solieria filiformis*	R-Phycoerythrin (R-PE)	0.025 M phosphate buffer, pH 6.5	0.14 mg/g dry weight (dw)	-	Phosphate buffer	360 min	4 °C	[151]
*Gracilaria gracilis*	Phycobiliproteins	Maceration and freeze–thaw	3.58 mg/g	-	Different phosphate buffer concentrations (0.01 M < C < 1 M, pH 6.8)	5–30 min	RT	[152]
*Gracilaria gracilis*	Phycobiliproteins	Ultrasound-assisted extraction (ultrasonic water bath and ultrasonic probe)	1.48–1.99 mg/g	-	Different phosphate buffer concentrations (0.01 M < C < 1 M, pH 6.8)	10 s–10 min	RT	[152]
*Gracilaria gracilis*	Phycobiliproteins	High pressure-assisted extraction	-	-	Different phosphate buffer concentrations (0.01 M < C < 1 M, pH 6.8)	5–30 min	RT	[152]
*Gracilaria gracilis*	Phycobiliprotein	Maceration	-	-	Phosphate buffer pH 6.8 (0.1 M)	10 min	RT	[153]
*Porphyra sensu lato*	Phycobiliproteins	Alkaline hydrolysis	4.29 ± 0.54 mg/g dry weight (dw) (PE), 1.24 ± 0.24 mg/g DW (PC)	-	5.25% sodium carbonate (SC)	-	80 °C	[154]
*Porphyra sensu lato*	Phycoerythrin (PE), phycocyanin (PC)	Enzymatic hydrolysis	12.20 ± 3.70 mg/g dry weight (dw) (PE), 6.71 ± 1.69 mg/g DW (PC)	-	Miura cocktail	122 min	27.66 °C	[154]
*Gelidium sesquipedale*	Phenolic compounds	Ultrasound-assisted extraction (UAE)	189.3 ± 11.7 mg GAE/100 g dw (40 °C, 15 min) and 205.6 ± 7.7 mg GAE/100 g dw (40 °C, 30 min)	81%	Ethanol/Water (50:50 *v*/*v*)	15 and 30 min	RT and 40 °C	[148]
*Porphyra sensu lato*	Phenolic compounds	Alkaline hydrolysis	3.19 ± 0.41 mg/g dry weight (dw) (1%, 3.53 ± 0.64 mmg/g dry weight (dw) (2.5%)	-	1% and 2.5% sodium carbonate (SC)	-	80 °C	[154]
*Porphyra sensu lato*	Phenolic compounds	Enzymatic hydrolysis	3.08 ± 0.22 mg/g dry weight (dw)	-	Miura cocktail	-	-	[154]

## 4. Conclusions and Outlook

This review provided an overview of the structures of neuroprotective compounds extracted from *red algae* over the past decade, including analyses of their structural properties and extraction methods, such as supercritical fluid extraction (SFE) and ultrasound-assisted extraction (UAE), which significantly enhance the separation efficiency of valuable compounds. The neuroprotective mechanisms of these described compounds against AD via targeted pathways were then deeply described. Finally, the overall neuroprotective mechanisms of these compounds were outlined to comprehensively extend the understanding of *red algae*-derived neuroprotectants: Sulfated polysaccharides (e.g., carrageenan, porphyran) suppress neuroinflammation via inhibition of the NF-κB pathway, protecting mitochondrial function, and enhancing BDNF expression to improve cognitive function. Phycobiliproteins and their degraded peptides scavenge reactive oxygen species (ROS) and activate antioxidant pathways. Carotenoids (e.g., lutein, zeaxanthin) ameliorate cognitive decline via inhibition of acetylcholinesterase and pro-inflammatory cytokines. Phenolic compounds (e.g., bromophenols) exert multi-target protection through dopaminergic system modulation and Nrf2 pathway activation. The use of emerging extraction technologies, such as microwave-assisted and enzyme-assisted methods, can serve to optimize the yield of bioactive compounds while preserving their bioactivity, although challenges remain in terms of defining molecular targets and standardizing extraction protocols.

Future research should prioritize elucidating the precise molecular targets of *red algal* compounds—such as identifying binding sites on the NLRP3 inflammasome or dopamine receptors—in order to enable mechanism-driven drug design. Additionally, efforts should focus on the development of green extraction protocols, such as deep eutectic solvent-based methods, as well as nanocarrier systems to improve blood–brain barrier penetration, thereby enhancing translational potential. Preclinical validation using human iPSC-derived neuronal models, clinical trials evaluating combination therapies with existing AD drugs, standardization of extraction processes, and establishment of quality control metrics (e.g., HPLC-ESI-QTOF-MS fingerprinting) are essential steps for the industrial-scale production of *red algae*-derived neuroprotectants. However, their clinical translation will require overcoming challenges such as ADME properties, blood–brain barrier permeability, disparities between preclinical animal models and human neurological systems, and the lack of reliable biomarkers. Fortunately, advances in modern histological technologies, integrated screening platforms, molecular docking techniques, and the development of novel nanocarrier delivery systems (e.g., encapsulating *red algal* polysaccharides) may help to overcome these challenges. These developments promote the translational process and offer new insights as research on marine drugs deepens. Overall, *red algal* extracts are positioned as promising new candidates for the treatment of AD.

## Figures and Tables

**Table 2:** Methods, dosages, and results of various activity experiments for porphyran and its derivatives.

Activities	Compounds	In Vitro or In Vivo	Methods	Doses	Results	References
Antioxidant activity	Porphyran	In vitro	Superoxide, hydrogen peroxide, and hydroxyl radical scavenging activity (DC-porphyrin) of porphyrin derived from color-changing seaweed.	10–1000 µg/mL	Superoxide scavenging activity (IC50: 415.9 μg/mL) and hydroxyl radical scavenging activity (IC50: 32.7 μg/mL).	[61]
Anti-inflammatory activity	Porphyran	In vivo	The effects of DC-porphyrin and a DC-porphyrin component F1 on the production of inflammatory mediators induced by LPS in mice.	100 mg/kg	Significantly reduced the levels of pro-inflammatory mediators NO and TNF-α.	[62]
	Porphyran	In vivo and in vitro	Oral or intraperitoneal injection of porphyrin to inhibit the progression of DSS-induced colitis in mice.	50 μg/mL	The levels of interferon-γ and interleukin-17 in T cells of the oral porphyrin group decreased. Inhibited T cell activation by suppressing dendritic cells and macrophages.	[63]
	Porphyran	In vivo and in vitro	Inhibit the up-regulation of costimulatory molecules and CCR7 expression in bmdc induced by LPS in vitro and in vivo.	0, 10, 25, 50, 100 μg/mL	Porphyrin is a very promising drug for the treatment of endotoxin-mediated inflammatory diseases.	[64]
	Porphyran	In vivo	The effects of porphyrin on inflammatory factors, IgA antibodies, and non-specific immune factors in mice infected with ETEC-K88.	70.54 ng/mL, 79.20 ng/mL, 10 mg/d	Reduced the levels of pro-inflammatory factors (MCP-1, TNF-a, IFN-g, IL-6), IgA, and NBT.	[65]
Neuroprotective activity	Oligo-Porphyran	In vivo	PI3K/Akt/Bcl-2 pathway; dopamine (DA) metabolism; behavioral deficits; pole test; traction test.	25, 50 mg/kg	Regulated the PI3K/Akt/Bcl-2 pathway; ameliorated neurobehavioral deficits.	[66]
	Porphyran and its derivatives	In vitro	MTT assay; Rhodamine123 using flow cytometry.	<1 mg/mL	Both AP and PP antagonized the weak toxicity of 6-OHDA on MES23.5 dopaminergic cells.	[67]
	Porphyran	In vivo	PYP pretreatment can prevent the decline in cell viability and the increase in GRP78 expression caused by glutamate exposure.	1 μg/mL	Blocking the NMDA receptor reduced the phosphorylation level of JNK. Down-regulation of GRP78 expression, β-galactosidase activity, and neuromutability increased.	[68]
Anti-inflammatory activity	Phycobiliproteins	In vivo	Algae bile protein and chlorophyll a were obtained by water extraction, and d-DWE was obtained by hot lysozyme digestion to evaluate inflammatory factors.	-	Reduced TNF-α, IL-6, and NO in LPS-stimulated RAW 264.7 cells, alleviated the induced acute inflammation.	[69]

**Table 3:** Classification and characteristics of phycobiliproteins.

Type	Color	Absorption Peaks (nm)	Fluorescence Properties	Existence Form	Representative Algae Species
R-Phycoerythrin,R-PE	Pink-red	495, 545, 565	Strong red fluorescence (~575 nm)	Disk-shaped hexamers	Porphyra (Nori)
R-Phycocyanin, R-PC	Blue	615, 650	Orange fluorescence (~647 nm)	α and β subunits form into trimers	Gracilaria
Allophycocyanin, APC	Blue-green	650, 660	Deep red fluorescence (~660 nm)	Hexamers or trimers	Chondrus (Irish moss)
